# Influence of Storage Conditions on the Quality, Metabolites, and Biological Activity of Soursop (*Annona muricata.* L.) Kombucha

**DOI:** 10.3389/fmicb.2020.603481

**Published:** 2020-12-04

**Authors:** Wee Ching Tan, Belal J. Muhialdin, Anis Shobirin Meor Hussin

**Affiliations:** ^1^Department of Food Science, Faculty of Food Science and Technology, Universiti Putra Malaysia, Serdang, Malaysia; ^2^Department of Food Technology, Faculty of Food Science and Technology, Universiti Putra Malaysia, Serdang, Malaysia; ^3^Halal Products Research Institute, Universiti Putra Malaysia, Serdang, Malaysia

**Keywords:** antioxidant activity, Halal, fermented tea, beverages and biological samples, health promoting

## Abstract

Kombucha is a slightly alcoholic beverage produced using sugared tea via fermentation using the symbiotic culture of bacteria and yeast (SCOBY). This study aimed to optimize the production of soursop kombucha and determine the effects of different storage conditions on the quality, metabolites, and biological activity. The response surface method (RSM) results demonstrated that the optimum production parameters were 300 ml soursop juice, 700 ml black tea, and 150 g sugar and 14 days fermentation at 28°C. The storage conditions showed significant (*P* < 0.05) effects on the antioxidant activity including the highest antioxidant activity for the sample stored for 14 days at 25°C in light and the highest total phenolic content (TPC) for the sample stored for 7 days at 4°C in the dark. No significant effects were observed on the antimicrobial activity of soursop kombucha toward *Escherichia coli* and *Staphylococcus aureus*. The microbial population was reduced from the average of 10^6^ CFU/ml before the storage to 10^4^ CFU/ml after the storage at 4 and 25°C in dark and light conditions. The metabolites profiling demonstrated significant decline for the sucrose, acetic acid, gluconic acid, and ethanol, while glucose was significantly increased. The storage conditions for 21 days at 25°C in the dark reduced 98% of ethanol content. The novel findings of this study revealed that prolonged storage conditions have high potential to improve the quality, metabolites content, biological activity, and the Halal status of soursop kombucha.

## Introduction

Kombucha is a slightly alcoholic beverage produced via the fermentation of sugared tea using symbiotic culture of bacteria and yeast (SCOBY) for 7–21 days (Leal et al., 2018). Kombucha has a slight sweet, sourly, and refreshing taste with high acceptability by the consumers worldwide (Teoh et al., 2004). Several studies reported that kombucha demonstrated biological activities such as antimicrobial, antioxidant, anticancer, antidiabetic, and anti-inflammatory activities (Villarreal-soto et al., 2018; Gaggìa et al., 2019; Ivanišová et al., 2019). The biological activity of kombucha has strong interaction with the SCOBY that is also known as “tea fungus.” The SCOBY contains symbiotic culture of yeast (*Brettanomyces*, *Zygosaccharomyces*, *Saccharomyces*, and *Pichia*) and acetic acid bacteria (*Acetobacter xylinum*) (Bellut et al., 2020). The yeast’s role is mainly to hydrolyze the sugar (sucrose) in the tea to glucose and fructose and convert it to ethanol, while the bacteria utilize the ethanol to produce acetic acid (Kulshrestha et al., 2013). The final product will have low pH and broad range of bioactive compounds with different biological activities (Teoh et al., 2004). Kombucha was reported as a rich source of different metabolites including organic acids (acetic and glucuronic), vitamins (B1, B2, and B12), and slight ethanol (Villarreal-Soto et al., 2019). In addition, phenolic compounds were reported to be found in black tea kombucha including gallic acid, caffeine, rutin, quercetin, and catechin (Barbosa et al., 2020).

Kombucha is traditionally produced using black tea and table sugar (sucrose) as the main ingredients. Thus, great numbers of studies were carried out to modify the substrates by adding fruit juices to enhance the biological activities and improve the flavor profile (Akbarirad et al., 2017; Yavari et al., 2018). Tropical fruit juices are rich in phenolic compounds and have the potential to increase the phenolic compound content and biological activities of fruit-based kombucha. In a recent study, snake fruit juice kombucha was fermented for 14 days and demonstrated high antidiabetic activity as determined in an *in vivo* study (Zubaidah et al., 2019). In another study, apple juice [150 ml/l (*v*/*v*)] was added to black tea to produce fruit-based kombucha, and the result was a significantly higher total phenol content compared to the control kombucha without apple juice (Liamkaew et al., 2016). Watawana et al. (2015) observed enhanced antioxidant activity and phenolic content for a coffee beverage fermented using kombucha SCOBY. In another study, kombucha SCOBY significantly enhanced the antioxidant activity of coconut water fermented for 7 days (Watawana et al., 2016). Soursop (*Annona muricata* L.) is an exotic tropical fruit that is found abundantly in the Southeast Asia region (Pinto et al., 2005). Soursop fruit is aromatic and juicy and has white flesh, creamy texture, pleasant characteristic, and sour taste (Lutchmedial et al., 2004). Soursop fruit was reported as a rich source of bioactive compounds such as acetogenins, alkaloids, and phenolic compounds (George et al., 2015). Ho et al. (2020) produced an alcoholic beverage with strong antioxidant activity using soursop juice and a combination of two starter culture including mushroom (*Pleurotus pulmonarius*) and yeast (*Saccharomyces cerevisiae*).

According to Villarreal-Soto et al. (2019), fermentation process conditions have a significant impact on the bioactive compounds in kombucha in relation to their biological activities. Thus, the optimization of fermentation conditions is very critical to produce kombucha that is rich in bioactive metabolites. In addition, the storage conditions including time, light, and duration can have a significant impact on the quality, metabolites, and the biological activities of kombucha. To the best of the authors’ knowledge, no studies were carried out to optimize the production of soursop kombucha and determine the effects of storage conditions on the final product. Therefore, the aim of this was to optimize the production of soursop kombucha and determine the effects of different storage conditions including temperature, light, and time on the quality, metabolites, and biological activities of soursop kombucha.

## Materials and Methods

### Materials

Fresh and ripen soursop (*Annona muricata* L.) fruits were purchased from NSK Trade City in Selayang, Selangor. The kombucha starter culture was obtained from the Food Bioprocessing Laboratory, Faculty of Food Science and Technology, Universiti Putra Malaysia (UPM). Table refined sugar of food grade was purchased from *Gula Prai*, Malayan Sugar Manufacturing Co. Berhad, Malaysia, and black tea from BOH, Malaysia.

### Sample Preparation

The soursop fruits were washed under running tap water, peeled, deseeded, and cut into small size cubes. The fruits (500 g) were mixed (1:1, *w*/*w*) with water using a blender (Kenwood, England) (Abbo et al., 2006). Black tea was prepared by adding 5 g of tea leaves in 1 l of boiling water and infused for 5 min. The soursop juice was pasteurized at 65°C for 30 min. The fruit and tea were mixed at different concentrations including 300:700, 500:500, and 700:300 ml (*v*/*v*). The mixtures were added into sterile glass jar and sugar was added at different concentrations including 50, 100, and 150 g/l (*w*/*v*). The sugared mixtures were inoculated with kombucha starter [1:10 (*w*/*w*)] and incubated for 7, 14, and 21 days at 28 ± 2°C. Samples of the soursop kombucha were collected at days 7, 14, and 21 to conduct analysis following a previous method (Zubaidah et al., 2018). The optimum condition for the production of soursop kombucha was based on parameters such as antioxidant activity [2,2-diphenyl-1-picrylhydrazyl (DPPH) and ferric reducing antioxidant power (FRAP)], antimicrobial activity (*Escherichia coli* and *Staphylococcus aureus*), and microbiological analysis [total plate count, yeast and mold, and availability of lactic acid bacteria (LAB)]. Optimized soursop kombucha was chosen for the storage study to determine the effects of temperature (4 and 25°C) and dark and light conditions on the physiochemical properties, metabolites, and biological activity of soursop kombucha. Samples were collected at days 7, 14, and 21 to carry out the different analyses. The samples were prepared in triplicate and subjected to freeze drying for 48 h (LaboGene, Denmark).

### Physicochemical Properties

The pH was measured by a calibrated electric pH meter (JENWAY 3505, Essex, England). The total soluble solids (TSS) were measured using a refractometer (Atago N1, Tokyo, Japan).

### Proton Nuclear Magnetic Resonance Metabolomics Analysis

The soursop kombucha freeze-dried samples (10 mg) were mixed with 0.375 ml of methanol-D4 and 0.375 ml of KH_2_PO_4_ buffer in D_2_O (pH 6.0) containing 1% TSP as internal standard for relative quantification of the identified metabolites. The mixture was vortexed for 1 min and sonicated at 30°C for 15 min in an ultra-sonicator (Branson, United States). The solution was centrifuged at 13,000 rpm for 10 min, and 600 μl of supernatant was transferred to a nuclear magnetic resonance tube for proton nuclear magnetic resonance (^1^H-NMR) analysis (Muhialdin et al., 2020b). Spectra were recorded at 25°C with frequency of 500 MHz on a Varian Unity INOVA 500 MHz Spectrometer (Varian Inc., CA). Each sample was subjected to 64 scans and recorded with an acquisition time of 193 s, with a pulse width of 10 ppm and a relaxation delay of 1 s. The spectra were automatically phased and bucketed using Chenomx software, with standard bins of δ 0.05 ranging from region δ 0.50 to 10.00. The analysis was required to remove residual methanol region (δ 3.28–3.33) and water region (δ 4.70–4.96). Two-dimensional ^1^H–^1^H J-resolved and was employed to identify metabolites. Partial least square analysis (PLS) and principal component analysis (PCA) were performed using SIMCA-P software (Umetrics AB, Umeå, Sweden).

### Free Radical Scavenging (DPPH) Assay

The free radical scavenging activity of soursop kombucha was evaluated by DPPH assay following the method done by Chu and Chen (2006). The soursop kombucha (25 μl) was mixed with 225 μl of 1 mmol/l DPPH solution in 96-well micro-titter plates and incubated in the dark at room temperature for 30 min before proceeding to the measurement of absorbance at 517 nm using a spectrophotometer (Shimadzu, UVmini-1240, Tokyo, Japan). The control was water and DPPH solution. The scavenging capacity of soursop kombucha was calculated as follows:

(1) $$\text{Scanvenging}\text{activity}\left(\%\right)=\frac{A_{\text{control}}-A_{\text{sample}}}{A_{\text{control}}}\times 100\left(1\right)$$

whereas *A*_*blank*_ was the control reading and *A*_*sample*_ was the sample reading.

### Ferric Reducing Antioxidant Power Assay

The antioxidant activity was measured by FRAP assay described by Benzie and Szeto (1999). FRAP reagent was prepared by mixing acetate buffer (300 mmol/l, pH 3.6), a solution of 10 μM 2,4,6-tripyridyl-s-triazine (TPTZ) in 40 mmol/l HCl, and 20 mmol/l FeCl_3_ at 10:1:1 (*v*/*v*/*v*). The reagent (300 μl) and kombucha (10 μl) were mixed thoroughly in 96-well micro-titter plates and incubated in dark condition for 30 min, and the absorbance was measured at 593 nm using a spectrophotometer (Shimadzu, UVmini-1240, Tokyo, Japan). The standard curve was prepared by ferrous sulfate solution (FeSO_4_⋅7H_2_O) with the range of concentration from 0.1 to 1.0 mmol/l. The FRAP reading was expressed as mmol Fe(II)/ml.

### Total Phenolic Content (TPC)

The TPC of soursop kombucha was measured following a previous method (Chu and Chen, 2006). Fermented soursop kombucha (0.05 ml) was mixed to 2 ml of 2% sodium carbonate and kept for 2 min. Folin–Ciocalteu reagent (0.1 ml) was then mixed with the solution and incubated for 30 min in the dark, and the absorbance was measured at 750 nm using a spectrophotometer (Shimadzu, UVmini-1240, Tokyo, Japan). The standard curve was plotted using gallic acid with the concentration range of 0–100 mmol/l, and TPC-value was expressed as μg GAE/ml (Muhialdin et al., 2019).

### Microbial Growth Inhibition

The antimicrobial activity of soursop kombucha samples was evaluated against pathogenic bacteria including *Escherichia coli* 0157:H7 and *Staphylococcus aureus* ATCC6538 using the microtiter plate assay (Muhialdin et al., 2020b). The sample (100 μl) was pipetted into the wells of microtiter plates and mixed with 100 μl of nutrient broth containing 10^6^ CFU/ml. The nutrient broth (200 μl) containing the tested bacteria was pipetted into the wells of microtiter plates as control. The plates were incubated at 37°C for 24 h. The growth of inhibition of targeted bacteria was measured at OD_600_ using the BioTek EL × 800 ELISA reader. The percentage growth of *E. coli* and *S. aureus* was calculated according to the following formula:

(2) $$\begin{array}{rcl} & & \mathrm{inhibition\%}\\ & & =\frac{\begin{array}{l} \left(24h\;\mathrm{negative}\;\mathrm{control}-24h\;\mathrm{negative}\;\mathrm{control}\right)\\ \;-(24h\;\mathrm{sample}-0h\;\mathrm{sample}) \end{array}}{0h\;\mathrm{negative}\;\mathrm{conrtrol}}\times 100 \end{array}$$

### Microbial Load Evaluation

Total plate counts of the soursop kombucha samples were done using the standard plate count procedure according to the Bacteriological Analytical Manual-Food and Drug Administration (BAM-FDA) protocol (Maturin and Peeler, 2001). Briefly, 1 ml of the samples was mixed with 9 ml of sterile 0.1% peptone water (10^–1^ dilution) followed by serial dilutions 10^–2^–10^–6^. A total of 100 μl of each dilution was inoculated into Plate Count Agar (PCA) (Merck, United States) and spread evenly using a sterile plate spreader. The agar dishes were incubated at 37°C for 24 h and then the colonies were counted and reported as colony-forming units/ml (CFU/ml).

Yeast and mold counts of the kombucha samples were done using the standard plate count procedure according to the BAM-FDA protocol (Maturin and Peeler, 2001) following the previous procedure: 100 μl of each dilution was inoculated into Malt Extract Agar (Oxoid) and spread evenly using a sterile plate spreader. The Petri dishes were incubated at 30°C for 72 h and then the colonies of yeast and mold were counted and reported as colony-forming units/ml (CFU/ml).

LAB count in the soursop kombucha samples was done following the standard according to the BAM-FDA protocol (Maturin and Peeler, 2001). Each dilution (100 μl) was inoculated into Petri dishes with De Man, Rogosa, and Sharpe (MRS) agar (Merck, United Kingdom) and spread evenly using a sterile plate spreader. The Petri dishes were incubated upside down at 37°C for 48 h in anaerobic jar, and the colonies were counted and reported as colony-forming units/ml (CFU/ml).

### Data Processing and One-Way Analysis of Variance Data Analysis

The data was analyzed using Minitab version 17 (Minitab, Inc., United States). The statistical differences between the samples and controls were evaluated by one-way analysis of variance (ANOVA) with Tukey’s multiple comparison to identify significant differences (*P* < 0.05) among means for all samples. Results were shown as the mean of three triplicates ± SD.

## Results and Discussion

### Optimization of Soursop Kombucha Production

**FIGURE 1:**
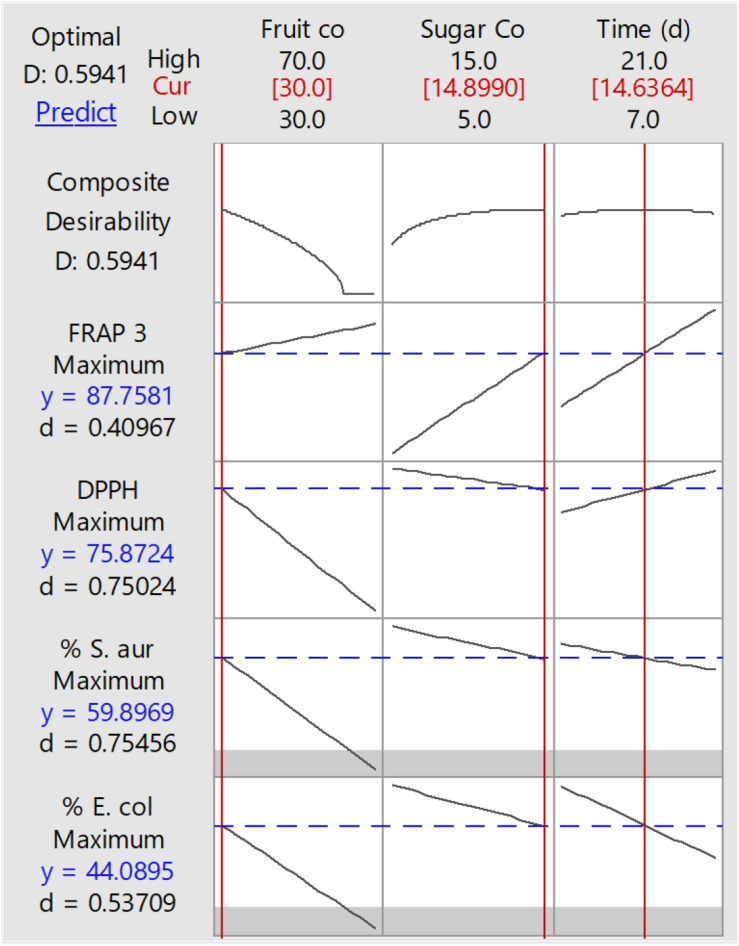
Response surface methodology (RSM) results of optimized conditions to produce soursop kombucha.

The response surface method (RSM) was carried out for 15 run orders, and the response was based on the microbial growth inhibition percentage against *Escherichia coli* and *Staphylococcus aureus*, DPPH, and FRAP for the different soursop kombuchas at different concentrations of fruit and sugar and storage time (Table 1). The predicted optimized conditions for soursop kombucha fermentation were found to be 300 ml soursop juice, 700 ml black tea, and 150 g sugar and 14 days fermentation at 28°C (Figure 1). The predicted conditions were validated, and no significant differences were observed between the actual and predicted conditions. The optimum conditions were applied to prepare the samples for the effect of storage study.

**TABLE 1:** Response surface methodology (RSM) of soursop kombucha at different concentrations of fruit and sugar and fermentation times.

Nos.	Fruit (%)	Sugar (%)	Time (days)	*E. coli* inhibition (%)	*S. aureus* inhibition (%)	DPPH (%)	FRAP [mmol Fe(II)/mL]
1	70	10	7	0	0	63.92	358.03
2	50	15	7	0	0	66.11	355.11
3	30	15	14	68.8	69.74	68.68	372.75
4	70	15	14	0	0	60.32	357.05
5	50	10	14	82.09	79.38	72.86	352.05
6	30	5	14	77.14	74.85	77.04	344.83
7	50	5	7	78.52	78.13	67.35	349.97
8	70	5	14	0	0	64.33	348.72
9	30	10	21	0	78.8	77.91	358.02
10	50	5	21	0	0	69.05	384.41
11	70	10	21	0	0	71.65	352.75
12	50	15	21	0	0	74.79	337.05
13	50	10	14	0	0	70.87	344.41
14	30	10	7	75.44	70.52	81.05	373.02
15	50	10	14	50.08	60.78	80.7	357.33

### ^1^H-NMR Identification for Metabolites Changes

The sucrose concentration was significantly (*P* < 0.05) reduced from day 7 until day 21 of storage at different conditions (Table 2). In comparison, fructose and glucose concentrations significantly increased in correlation with the storage time. The observed fructose and glucose changes were due to the breakdown of sucrose by the bacteria and yeasts in the soursop kombucha into monosaccharides. Villarreal-soto et al. (2018) reported that sucrose is hydrolyzed into glucose and fructose during the fermentation due to the enzymatic activity of different microorganisms including yeast and bacteria. The hydrolysis is catalyzed by the enzyme invertase secreted from yeasts and bacteria into the fermentation substrates (Rasu Jayabalan et al., 2014). However, fructose concentration was significantly lower compared to glucose at all the storage conditions (Table 2). The difference is may be due to the preference of the yeast cells to rapidly utilize fructose as source of energy (Neffe-skocińska et al., 2017). In this study, the sugar concentration changes are mainly due to the activity of the soursop kombucha microorganisms that originated from the SCOBY that was used for the fermentation process (Leal et al., 2018).

**TABLE 2:** The effect of different storage conditions on the major metabolite changes of soursop kombucha samples and their concentration (mmol/l) as determined using ^1^H-NMR metabolomics-based analysis.

Sample	Sucrose δ 5.40 (d)	Fructose δ 3.823 (m)	Glucose δ 4.58 (d)	Acetic acid δ 1.98 (s)	Malic acid δ 2.7 (d)	Gluconic acid δ 4.18 (d)	Ethanol δ 1.17 (t)
Control (0 h)	11.51	21.01	4.60	0.33	0.13	6.59	3.28
7RL	1.01	13.39	13.05	0.15	0.12	1.11	1.02
7RD	1.12	13.67	17.21	0.11	0.12	1.00	1.00
7CL	1.06	13.77	16.19	0.16	0.10	1.13	1.04
7CD	1.16	13.90	18.70	0.22	0.12	1.13	1.02
14RL	0.81	14.43	19.65	0.23	0.12	0.90	1.07
14RD	0.81	18.12	20.61	0.34	0.13	1.03	1.95
14CL	1.00	16.37	20.62	0.18	0.12	1.19	0.45
14CD	1.05	17.04	20.47	0.23	0.13	1.17	0.67
21RL	0.54	16.29	25.92	0.64	0.11	0.66	0.14
21RD	0.19	15.86	25.56	0.63	0.11	0.28	0.06
21CL	0.01	15.31	24.41	0.07	0.12	0.70	0.37
21CD	0.01	15.15	23.07	0.07	0.11	0.71	0.26

*7, 14, and 21 days are storage times.R, room temperature; C, chilled temperature; L, light condition; D, dark condition.*

On the other hand, acetic acid, malic acid, and gluconic acid were all present in soursop kombucha (Table 2). A previous study reported that the chemical compositions in kombucha include organic acids such as acetic acid, malic acid, and gluconic acid (Ramachandran et al., 2006). Acetic acid in soursop kombucha samples increased from days 7 to 21 for samples stored under room temperature. The increased content is due to the presence of *Acetobacter* bacteria in the soursop kombucha samples that produce acetic acid. Acetic acid bacteria are the dominant aerobic microorganisms in kombucha that utilize alcohol as substrate to produce acetic acid. In the presence of oxygen, acetic acid bacteria have the ability to keep producing acetic acid in the soursop kombucha beverage (Villarreal-soto et al., 2018). Cvetković et al. (2008) reported that acetic acid bacteria function to produce a new cellulose layer and metabolize ethanol to produce organic acids. Acetaldehyde is converted into ethanol, whereas acetaldehyde hydrate is being converted into acetic acid facilitated by the enzyme acetaldehyde dehydrogenase (Jayabalan et al., 2007). On the other hand, malic acid content was stable in all soursop kombucha along the storage period at different conditions. Malic acid is one of the dominant organic acids that can be found commonly in kombucha (Jayabalan et al., 2007). In addition, gluconic acid concentration decreased in correlation to the storage period from 0 h to day 21. Gluconic acid is produced by *Gluconobacter* bacteria which prefer the utilization of glucose during fermentation (Rasu Jayabalan et al., 2014). In another study, *Komagataeibacter xylinus* was reported as the dominant species in *Acetobacter* that acts to oxidize glucose to gluconic acid (Villarreal-soto et al., 2018). Gluconic acid is one of the healthy promoting metabolites found in kombucha made up of sucrose and black tea under optimized fermenting conditions (Chen and Liu, 2000; Malbaša et al., 2002; Jayabalan et al., 2007). Gluconic acid is converted to 2-ketogluconate and finally to 2,5-diketogluconic acid during oxidation by gluconic acid dehydrogenase and 2-ketogluconate dehydrogenase enzymes, respectively (Ramachandran et al., 2006).

**FIGURE 2:**
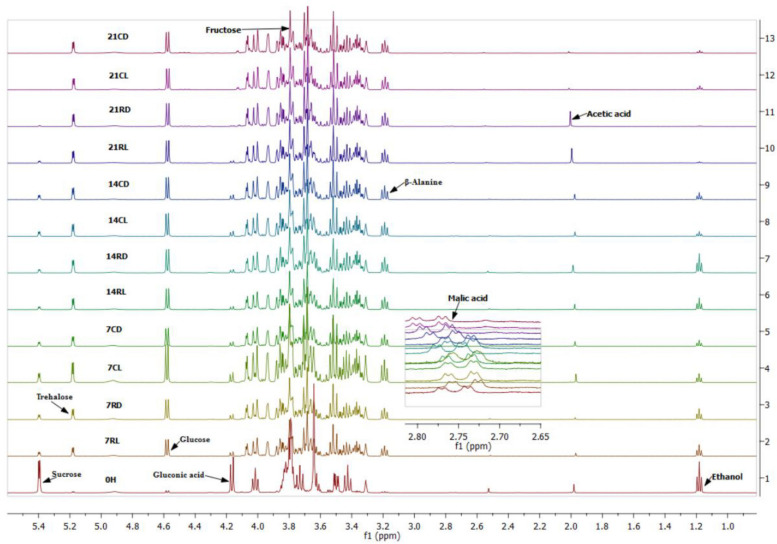
Proton nuclear magnetic resonance (^1^H-NMR) spectra of soursop kombucha at different storage conditions. Full spectra of δ 1.0–5.5 ppm. Identified ^1^H-NMR signals: sucrose, fructose, glucose, acetic acid, malic acid, gluconic acid, and ethanol. 7, 14, and 21 days are storage times. R, room temperature; C, chilled temperature; L, light condition; D dark condition.

The ethanol concentration in soursop kombucha samples gradually declined from 0 h until day 21 (Table 2). The sample at 0 h showed the highest ethanol concentration of 3.284 mmol/l, whereas the sample 21RD stored for 21 days at room temperature in the dark showed the lowest ethanol concentration (0.062 mmol/l) (Figure 2). Ethanol is produced by the yeast in kombucha from the sugar substrates via the glycolysis pathway, as sucrose is hydrolyzed into glucose and fructose and catalyzed by an enzyme secreted by yeasts. Ethanol produced during the fermentation process can be converted into acetic acid by acetic acid bacteria (Villarreal-soto et al., 2018). Acetic acid bacteria will then reduce the ethanol concentration via utilizing ethanol as source of carbon (Battikh et al., 2012). The observed reduction in the ethanol content indicated that different storage conditions can improve the quality of soursop kombucha for the concerned religious consumers. Beverages containing high ethanol concentrations are called non-Halal according to the Islamic regulations, as ethanol is limited to less than 1% (Alzeer and Abou Hadeed, 2016). The best storage conditions to decrease ethanol were 21 days, room temperature, and in dark storage. These storage conditions are recommended in this study to reduce ethanol content for Muslim consumers.

### Antioxidant Activities

Bioactive compounds present in fermented foods and beverages have the key role for the antioxidant activity. The soursop kombucha exhibited good antioxidant activity at all the storage conditions for days 7, 14, and 21 (Table 3). The scavenging ability of DPPH for soursop kombucha declined in the following order: 14RL > 7CD > 7RL > 14RD > 14CL > 7RD > 14CD > 7CL > 21CL > 21RL > 21CD > 21RD. Soursop kombucha stored for 14 days at room temperature and exposed to light conditions (14RL) exhibited the highest antioxidant activity (90.76%), while 21 days at room temperature and dark conditions (21RD) showed the lowest antioxidant activity (85.71%). The results did not show any significant differences in DPPH radical scavenging assay (*P* ≥ 0.05). The DPPH inhibition percentage was 90% and above which indicates high antioxidant activity exhibited by soursop kombucha at all the storage conditions. The SCOBY plays an important role in affecting the composition of the produced kombucha due to the unique microflora present in different SCOBY (Chen and Liu, 2000). Another study reported that the utilization of different SCOBY as starter in kombucha resulted in variable antioxidant activities (Malbaša et al., 2011). The findings of this study agree with the previous studies as modifying the conditions showed significant impact on the antioxidant activity.

**TABLE 3:** The antioxidant activity measured using DPPH, FRAP, and TPC assays of soursop kombucha during the storage at different conditions.

Sample	DPPH (%)	FRAP [mmol Fe(II)/ml]	TPC (μg GAE/mL)
7RL	89.12 ± 0.04^ab^	430.25 ± 0.02^a^	39.55 ± 0.25^bc^
7RD	88.46 ± 0.04^ab^	424.56 ± 0.01^a^	41.99 ± 0.12^abc^
7CL	87.56 ± 0.03^ab^	410.67 ± 0.01^a^	46.94 ± 0.07^a^
7CD	89.22 ± 0.03^ab^	414.83 ± 0.01^a^	46.96 ± 0.07^a^
14RL	90.76 ± 0.01^a^	429.83 ± 0.01^a^	37.90 ± 0.07^c^
14RD	88.98 ± 0.01^ab^	425.11 ± 0.02^a^	37.97 ± 0.08^c^
14CL	88.88 ± 0.02^ab^	419.00 ± 0.01^a^	39.14 ± 0.06^bc^
14CD	87.98 ± 0.03^ab^	434.69 ± 0.00^a^	38.81 ± 0.10^c^
21RL	86.12 ± 0.02^ab^	422.89 ± 0.00^a^	42.21 ± 0.11^abc^
21RD	85.71 ± 0.02^b^	420.67 ± 0.01^a^	43.07 ± 0.15^abc^
21CL	87.15 ± 0.02^ab^	419.14 ± 0.01^a^	41.69 ± 0.13^abc^
21CD	86.02 ± 0.03^ab^	432.80 ± 0.04^a^	45.56 ± 0.13^ab^

*Data are expressed as mean ± SD of three replicates (n = 4). Means with different superscript letters in columns show significant difference using Tukey’s test (P < 0.05) for different storage conditions. 7, 14, and 21 days are storage times.R, room temperature; C, chilled temperature; L, light condition; D, dark condition.*

The sample 14CD showed the highest FRAP-value of 434.69 μl GAE/ml, while 7CL has the lowest FRAP-value of 410.67 ± 0.013 mmol Fe(II)/ml (Table 3). The result showed that the prolonged storage has no significant effects on the FRAP-values (*P* ≥ 0.05) (Table 3). However, the results revealed that all soursop kombuchas have high antioxidant properties as the results expressed high ferric reducing antioxidant power, with a range of 410.67–434.69 mmol Fe(II)/ml. The results of the FRAP assay are based on reducing Fe^3+^ in ferric-tripyridyltriazine to Fe^2+^, and it is commonly used to evaluate antioxidant activity (Kim et al., 2013). A previous study reported that the lipophilic antioxidant is the main antioxidant compounds in soursop pulp with hydrogen donation as the mechanism of action (Gordillo et al., 2012). The strong antioxidant activity may be a result of the bioactive metabolites from soursop juice that contributed to the antioxidant property of soursop kombucha. The results showed that the soursop kombucha stored at different conditions had different TPC concentrations. In comparison to all samples, TPC in sample 7CD was the highest measuring at 46.96 μg GAE/ml. There was only a slight difference observed between the samples. The result showed no significant changes in the TPC (*P* ≥ 0.05). Phenolic compounds perform antioxidant activity and are recognized for their function in reducing free radical activity and oxidative stress. Yeasts and bacteria in SCOBY produce an enzyme that acts in the conversion of polyphenolic complex into simpler phenolic components. The increased phenol content could be due to biotransformation that modifies a specific functional group into composing substances facilitated by enzymes. Enzymes are used in biotransformation to escalate specific biological activities (Srihari and Satyanarayana, 2012). Bhanja et al. (2009) reported phenol as one of the organic compounds that has a strong correlation to the antioxidant activity: the greater the amount of phenols being produced during fermentation as metabolites, the greater is the antioxidant activity. In a previous study, prolonged fermentation periods increased the TPC and antioxidant activity with no effects on the pH-value and the sensory characteristics (Muhialdin et al., 2019).

### Antimicrobial Activity

The highest percentages of microbial growth inhibition against *E. coli* and *S. aureus* were 99.83 and 100.00% (Table 4). The results showed that storage conditions have no significant (*P* ≥ 0.05) effects on the antimicrobial activity of soursop kombucha. The results indicated the stability of the strong antimicrobial activity of soursop kombucha at prolonged storage due to the presence of organic acids (Muhialdin et al., 2020a). In a previous study, kombucha demonstrated antibacterial activity toward a broad range of pathogens due to the presence of organic acids (Greenwalt et al., 1998). The results of this study showed similar findings as acetic acid was found at high concentrations in the soursop kombucha. Acetic acid has the ability to penetrate into gram-positive bacteria cells more easily than gram-negative bacteria due to its lipophilic characteristics (Naidu, 2000). Protons are released when acetic acid undergoes disassociation, and this causes the increment in acidity. The cell membrane function of targeted bacteria will be disrupted once the contact is established with the protons. Acetic acid denatures enzyme activity and disrupts the permeability of the cell membrane. Acetic acid in soursop kombucha is therefore affecting the antimicrobial activity of this fermented beverage. Dufresne and Farnworth (2000) observed that ethanol and acetic acid contents inhibit the growth of pathogens and are related with antimicrobial activity in their study. In another study, the antimicrobial activity of kombucha was reported against *Candida* spp. as compared with the common black tea that showed low antimicrobial activity (Battikh et al., 2012).

**TABLE 4:** The effects of different storage conditions on the antimicrobial activity of soursop kombucha toward *Escherichia coli* and *Staphylococcus aureus* expressed as growth inhibition percentage.

	Microbial growth inhibition (%)	
Samples	*Escherichia coli*	*Staphylococcus aureus*
7RL	99.83 ± 0.00^a^	98.41 ± 0.00^f^
7RD	99.57 ± 0.00^a^	98.14 ± 0.00^de^
7CL	99.65 ± 0.00^a^	98.76 ± 0.00^a^
7CD	99.91 ± 0.00^a^	99.56 ± 0.00^bc^
14RL	98.96 ± 0.03^a^	99.65 ± 0.02^bc^
14RD	99.57 ± 0.00^a^	98.58 ± 0.00^ef^
14CL	95.66 ± 0.00^a^	100.00 ± 0.00^a^
14CD	99.74 ± 0.00^a^	98.58 ± 0.00^def^
21RL	99.13 ± 0.00^a^	98.14 ± 0.00^bc^
21RD	99.57 ± 0.00^a^	98.50 ± 0.00^b^
21CL	99.13 ± 0.00^a^	97.70 ± 0.00^b^
21CD	98.78 ± 0.010^a^	97.70 ± 0.001^b^

*Data are expressed as mean ± SD of three replicates (n = 4). Means with different superscript letters in columns show significant difference using Tukey’s test (P < 0.05) for different storage conditions. 7, 14, and 21 days are storage periods.R, room temperature; C, chilled temperature; L, light condition; D, dark condition.*

### Microbiological Analysis

The microbial load for soursop kombucha was determined for the total plate count, yeast, and LAB counts (Table 5). The results showed that all microorganisms were decreasing during the 21 days of storage for all the conditions. According to Fifteenth schedule, Regulation 39 on the Microbiological Standard in Food Regulation 1985, the maximum level of total plate count has to be at or less than 10^5^/ml for ready-to-eat foods and beverages. The results showed that the aerobic bacteria count significantly declined from 7 (2.98 × 10^6^ CFU/ml) to 21 days (2.80 × 10^4^ CFU/ml). The result indicated that prolonged storage can enhance the safety of the soursop kombucha. The microbial load declined to 10^5^ after storage for 14 days which is highly safe for the consumers. The reduction of the aerobic bacteria could be due to the high acidic environment and the depletion of the nutrients. Watawana et al. (2015) reported that the increased acidity during storage of fermented beverages reduces oxygen content and the number of aerobic bacteria viable cells. The results of this study agreed with the previous study, and the pH of the soursop kombucha declined during the prolonged storage. The count for yeast was decreasing during the storage period, significantly for the samples exposed to light at room temperature for 21 days storage (Table 5). The reduction in yeast count could be due to the reduction in sugar concentration that is required for yeast growth and cell production. Abbo et al. (2006) reported that low sugar concentration, low pH, and storage at 28°C limited the growth of yeast in soursop juice. The effects of light exposure and storage temperature exhibited unexpected effects on the availability of LAB cells. The exposure to light significantly reduced the cell count for the LAB to 3.40 × 10^4^ at room temperature and 3.70 × 10^4^ at chilled temperature. According to Trisnawita et al. (2018), the storage condition at chilled temperature (4°C) showed no significant effects on the LAB cells after prolonged storage for 28 days. Thus, the availability of LAB significantly declined at 28°C for 28 days. In another study, a beverage containing LAB stored at 25°C showed significant reduction in the LAB cell count (Begum et al., 2015). The reduction of LAB cell count in beverages was attributed to several reasons including dehydration of cells, high water activity, low pH-value, and depletion of the nutrients in the substrate (Trisnawita et al., 2018). Nevertheless, the LAB cell count showed no strong interaction with storage temperature, but an interaction was observed for light exposure.

**TABLE 5:** The effect of different storage conditions on the microbiological load in the soursop kombucha.

Soursop kombucha	Total plate count (CFU/ml)	Yeast count (CFU/ml)	Lactic acid bacteria count (CFU/ml)
7RL	2.24 × 10^6^	2.91 × 10^5^	1.39 × 10^6^
7RD	2.98 × 10^6^	2.04 × 10^6^	2.12 × 10^6^
7CL	2.61 × 10^5^	9.80 × 10^5^	4.00 × 10^5^
7CD	2.90 × 10^5^	9.10 × 10^5^	2.20 × 10^5^
14RL	1.35 × 10^5^	4.10 × 10^5^	2.90 × 10^5^
14RD	1.18 × 10^5^	1.50 × 10^5^	1.20 × 10^5^
14CL	1.30 × 10^5^	1.20 × 10^5^	2.40 × 10^5^
14CD	2.95 × 10^5^	3.60 × 10^5^	4.60 × 10^5^
21RL	2.80 × 10^4^	3.70 × 10^4^	3.40 × 10^4^
21RD	7.20 × 10^4^	1.63 × 10^5^	1.03 × 10^5^
21CL	9.30 × 10^4^	6.30 × 10^4^	3.70 × 10^4^
21CD	1.20 × 10^5^	1.59 × 10^5^	1.59 × 10^5^

*Data are expressed as mean ± SD of three replicates (n = 4). Means with different superscript letters in columns show significant difference using Tukey’s test (P < 0.05) for different storage conditions. 7, 14, and 21 days are storage times.R, room temperature; C, chilled temperature; L, light condition; D, dark condition; CFU, colony-forming unit.*

## Conclusion

This is the first study to develop and optimize the production of soursop kombucha. The developed beverage showed strong antioxidant and antimicrobial activities and high phenolic content. The different storage conditions demonstrated slight effects on the biological activities and significant effects on the metabolites of the soursop kombucha. Sucrose was significantly declined and glucose was significantly increased. The storage of soursop kombucha for 21 days at room temperature in dark conditions degraded 98% of the ethanol content. The microbial load for aerobic and anaerobic bacteria and yeast showed significant decline and high interaction with light exposure. The results revealed that prolonged storage for 21 days has high potential to improve the quality and metabolite content for soursop kombucha. Moreover, storing soursop kombucha for 21 days at room temperature with dark conditions can significantly improve the Halal status for consumers with religious concerns and allergies to alcohols. Further study is highly recommended to determine the consumer preference and acceptability for commercialization of soursop kombucha.

## Data Availability Statement

The original contributions presented in the study are included in the article/supplementary material, further inquiries can be directed to the corresponding author/s.

## Author Contributions

WT performed the fermentation experiments, analyses, and statistics and wrote the manuscript. BM designed the experiments, performed NMR analysis, bioinformatics analysis, and reviewed the manuscript draft. AM supervised the project. All authors checked and approved the manuscript.

## Conflict of Interest

The authors declare that the research was conducted in the absence of any commercial or financial relationships that could be construed as a potential conflict of interest.
